# Evaluation of the integration of social accountability values into medical education using a problem-based learning curriculum

**DOI:** 10.1186/s12909-022-03245-6

**Published:** 2022-03-16

**Authors:** Nihar Ranjan Dash, Mohamed H. Taha, Sarra Shorbagi, Mohamed Elhassan Abdalla

**Affiliations:** 1 Clinical Sciences Department, College of Medicine, University of Sharjah, 27272 Sharjah, United Arab Emirates; 2 College of Medicine and Medical Education Center, University of Sharjah, Sharjah, United Arab Emirates; 3 Department of Community and Family Medicine and Behavioural Sciences, College of Medicine, University of Sharjah, Sharjah, United Arab Emirates; 4 School of Medicine, University of Limerick, Limerick, Ireland

**Keywords:** Social accountability, Medical education, Problem-based learning, Case scenarios

## Abstract

**Background:**

Medical schools have the obligation to direct their education toward addressing the priority health concerns of the societies that they serve. The purpose of this study was to evaluate the integration of the concepts and values of social accountability into the case scenarios that are used in a problem-based learning (PBL) curriculum at a medical school in the United Arab Emirates (UAE).

**Methods:**

A validated “social accountability inventory for PBL” was used for examining 70 case scenarios in a problem-based learning (PBL) medical curriculum.

**Results:**

The findings of the study showed that patient gender and age were included in all the 70 case scenarios. Vast majority of the case scenarios had successfully integrated the social accountably values in addressing the following: the major health problems or social health concerns of the UAE (73%), the social determinants of health (70%), the contextual integration of medical professionalism (87%), the evolving roles of doctors in the health system (79%), the healthcare referral system based on the case complexity (73%), the involvement of different stakeholders in healthcare (87%), psychosocial issues rather than only the disease-oriented issues (80%) and the values of health promotion/prevention (59%) cases. On the other hand, the case scenarios were deficient in integrating other social accountability values that related to the importance of treatment cost-effectiveness (91%), consideration of the underserved, disadvantaged or vulnerable populations in the society (89%), patient’s ethnicity (77%), multidisciplinary approach to patient management (67%), the socioeconomic statuses of patients (53%), the issues regarding the management of the health system (39%) respectively. There was variability in integrating the social accountability values in case scenarios across different units which are based on organ system.

**Conclusion:**

Medical educators and healthcare leaders can use this valuable data to calibrate the curriculum content, especially when using a problem-based learning curriculum to integrate the values of social accountability such as relevance, quality, equity and cost-effectiveness to train the future generation of healthcare providers to be ready to address the ever-changing and diverse needs of the societies.

## Background

According to the World Health Organization, the concept of social accountability obligates medical schools to direct their activities to addressing the priority health needs of the societies, and the stakeholders and partners for health, including the public, should jointly define those needs and concerns [1]. Medical schools worldwide educate and train their workforces to serve the needs of the societies and to shape the healthcare systems. Therefore, in terms of their plans, actions and impacts, medical schools are accountable to the societies that they serve. The demand for these schools to demonstrate social accountability is on the rise from legislation, regulation, and accreditation bodies [2]. Medical schools are expected to document both social accountability plans in their organization functions and social accountability actions in their education and research to exhibit the positive impacts these have on their health systems, communities, regions and nations [3].

Several tools, grids and frameworks have been developed to measure the integration of social accountability concepts into medical education at institutional levels [4, 5]. In addition, a number of medical schools in the Middle-Eastern region and worldwide have used these tools to assess compliance with the concepts of social accountability [6–8]. However, although these instruments have helped to clarify this concept and its definition, therefore reducing ambiguities and promoting consistency in the application of social accountability in medical education, none of these instruments has helped in assessing the needs of curricula to support the medical schools in becoming socially accountable [9]. Like the rest of the world, the medical schools in the United Arab Emirates (UAE) need to adapt and make necessary changes and adjustments in their education, research and service programs according to the societal health needs to demonstrate accountability. To become more responsive to their societies and national healthcare requirements, individual medical schools need to assess how effectively they integrate social accountability into their teaching curricula. This will help them to update and address the changing priorities of their community healthcare needs, expose students to learning opportunities that address the societal health needs and, finally, produce healthcare professionals who have the knowledge and skills to handle the priority health needs of the communities they intend to serve.

The UAE, with a population of 9.9 million, has a unique mix of expatriates (88%) and native Emiratis (12%) [10]. Migrant labourers comprise about 90 percent of the UAE workforce and among these the construction workers, domestic workers, illegal immigrants are considered vulnerable population. Similar other societies, social inequality does exit in UAE that include income gap, gender inequality, health care, and social class [11]. With the constant influx in recent two decades of a population from other countries, there has been a considerable change in the lifestyles and healthcare needs of those in the UAE. Currently, the most common causes of death and disability are non-communicable diseases (77%) such as cardiovascular diseases, road injuries, stroke, diabetes, chronic kidney diseases, chronic obstructive pulmonary diseases, depressive disorders, drug use disorders, pancreatic cancer and self-harm. Communicable diseases such as tuberculosis, pneumonia, hepatitis and infections account for approximately 12% of deaths and disabilities [12, 13]. To serve the healthcare needs of its population, the UAE has seven medical schools that graduate thousands of healthcare professionals annually, including doctors, nurses and other healthcare workers [4].

The aim of this study, then, was to evaluate the integration of the concepts and value of social accountability into the case scenarios used in a problem-based learning (PBL) curriculum in medicine.

## Methods

The study was performed after obtaining the necessary institutional ethics approval (REC-19-01-26-01).

## Study setting

The College of Medicine at the University of Sharjah (COMUOS) in the UAE has a medical education program that extends over six years and adopts a student centred problem-based learning curriculum. The medical curriculum is further divided into three phases: phase I—foundation year, phase II—pre-clerkship phase (years 1, 2 and 3) and phase III—clerkship phase (years 4 and 5). In phase II, the curriculum is themed around organ systems and structured into nine units such as cardiovascular, musculoskeletal, neurosciences, respiratory, genitourinary etc.

During this phase, a total of 70 written case scenarios simulating real patients’ problems constitute the main learning and teaching strategy of the problem-based learning (PBL) curriculum. Year 1 and 2 have 24 cases each and year 3 has 22 cases. Year 4 and 5 are clerkship years during which the students rotate in different clinical disciplines in hospitals. Paper based case scenarios are now replaced with real patients.

The PBL case scenarios are the driving engine of learning and teaching are designed to generate learning objectives or triggers that will help the student to acquire knowledge and understanding.

The scenarios are written in such a way that it will lead students to a particular area of study to achieve those learning objectives and ensure that all students are exposed to the same problems.

The case scenarios are implemented through a typical PBL cycle [14, 15] comprising of a small group of students (usually 8 to 10) and a facilitator who facilitates the session. The first session is held at the beginning of the week and the second session is at the end of the week [15]. Both sessions are of two hours each. In the first session the case scenario helps in defining the problem, generating triggers and objectives. It helps brainstorming, discussion and creating hypothesis. In the second session, the case scenario help in understanding the concepts, application and creation of concept maps. Between the two sessions students are involved in different activities such as resource session or lecture, practical and laboratory session, clinical skill session, group discussion and self-directed learning session to support their learning and achieving the objectives of the problem.

The PBL case scenarios were written by a team of subject matter experts, based on the outcome objectives of the curriculum, unit and disciplines. The case scenarios are selected keeping relevance to the local community and healthcare system. They contain cues to stimulate discussion and encourage students to seek explanations for the issues presented. The case scenarios are reviewed and updated annually based on faculty and student's feedbacks. Finally, the case scenarios are developed appropriate to the stage of the curriculum and the level of the students' understanding [16].

## Design

Evaluation of the social accountability concepts integration into case scenarios used in the problem-based learning curriculum was performed using a validated inventory “social accountability inventory for PBL” [17] . This inventory has 17 close-ended statements distributed under the following themes (social accountability values): relevance (10 questions), equity (5 questions), cost-effectiveness (1 questions) and quality (1 question) See appendix.

The four themes or values of social accountability are derived from World Health Organizations’ social accountability grid 1995 outlined as: (1) relevance (the extent to which medical education's goals align with the needs and aspirations of the communities it serves); (2) equity (making health care available to all, and medical education accessible to women and members of racial, ethnic, and other minorities); (3) cost-effectiveness (the contribution of medical education to cost-effective care); and (4) quality (the extent to which medical education contributes to high-quality, effective care, both in the settings where trainees receive care and in the future practice of graduates ) [1, 18].

The 10 questions examining relevance and the questions examining quality and cost-effectiveness collected responses on a 4-pont-Likert scale in numerical values: A (agree), B (disagree), C (neither agree nor disagree), and D (not applicable). The five questions of equity can be responded as yes, no or not applicable. Online data collection form was made using Survey Monkey® software. [https://www.esurveyspro.com/Survey.aspx?id=26158e7a-6ff9-4c9a-a9e8-f0452432d608]. Each PBL case scenario was evaluated by two assessors for the integration of social accountability concepts using the aforesaid inventory. In case of disagreement a third assessor was consulted and finally the decisions were made by consensus.

## Results

A total of 70 case scenarios used in the problem-based learning curriculum were evaluated for the integration of SA concepts. Table 1 shows the presence of these social accountability values in the case scenarios. In the relevance domain, the current study showed that the majority of case scenarios at COMUOS (73%, 51 of 70) had addressed the major health problems or social health concerns of the country [19, 20], 70% of the case scenarios (49 of 70) had triggers addressing one or more social determinants of health.

**Table 1:** Social accountability values present in the PBL case scenario

Q	Domain	Item	Agree *n*=70 (%)	Disagree *n*=70 (%)	Neither Agree or Disagree *n*=70 (%)	Not applicable *n*=70 (%)
1	Relevance	The problem case scenario is relevant to social health concerns.	51 (72.86%)	16 (22.86%)	3 (4.29%)	0 (0%)
2		The problem case scenario addresses one or more of the social determinants of health.	49 (70.00%)	19 (27.14%)	2 (2.86%)	0 (0%)
3		The problem case scenario points out the relevant values of health promotion and preventive measures.	41 (58.57%)	21 (30.00%)	2 (2.86%)	6 (8.57%)
4		The problem case scenario reflects the involvement of different stakeholders in health care.	61 (87.14%)	9 (12.86%)	0(0%)	0(0%)
5		The problem case scenario integrates the relevant psychosocial issues, rather than only disease-oriented issues.	56 (80.00%)	13 (18.57%)	1 (1.43%)	0 (0%)
6		The problem case scenario reflects the relevant health system management issues.	34 (48.57%)	27 (38.57%)	9 (12.86%)	0 (0%)
7		The problem case scenario includes the relevant elements of medical professionalism	61 (87.14%)	9 (12.86%)	0(0%)	0 (0%)
8		The problem case scenario includes triggers embedded in the (primary to tertiary) health care referral system based on the case complexity.	51 (72.86%)	18 (25.71%)	0 (0%)	1 (1.43%)
9		The problem case scenario includes triggers linked to the evolving roles of doctors in the health system	55 (78.57%)	12 (17.14%)	3 (4.29%)	0 (0%)
10		The problem case scenario includes triggers highlighting the importance of a multidisciplinary approach to patient management	20 (28.57%)	47 (67.14%)	3 (4.29%)	0 0%
11	Equity	The problem case scenario addresses the ethnicity of the patient.	16 (22.86%)	54 (77.14%)	0 (0%)	0 (0%)
12		The problem case scenario addresses the socioeconomic aspects of the patient.	33 (47.14%)	37 (52.86%)	0 (0%)	0 (0%)
13		The problem case scenario addresses the patient’s age group	69 (98.57%)	1 (1.43%)	2 (0%)	0 (0%))
14		The problem case scenario addresses the patient’s gender	70 (100%)	0(0%)	0 (0%)	0 (0%)
15		The problem case scenario includes under-served, disadvantaged, or vulnerable populations in society	8 (11.43%)	62 (88.57%)	0 (0%)	0 (0%)
16	Cost-effectiveness	The problem case scenario includes triggers for discussing treatment costs and providing alternatives	5 (7.14%)	64 (91.43%)	1 (1.43%)	0 (0%)
17	Quality	The problem case scenario includes the concept of ‘person-cantered healthcare’.	54 (77.14%)	10 (14.29)	4 (5.71)	2 (2.86%)

Similarly, 87% case scenarios (61 of 70) reflected contextual integration of medical professionalism and involvement of different stakeholders in healthcare. 56 of 70 case scenarios (80%) integrated the relevant psychosocial issues rather than only disease-oriented issues in their content. In 55 of the 70 cases scenarios (79%), evolving roles of doctors in the health system was mentioned and in 51 of the 70 case scenarios (73%) healthcare referral system based on the case complexity was stated. In the same relevance domain, a lesser number 41 cases (59%) applied the values of health promotion/prevention measures in their management plan, 34 cases (49%) reflected health system management issues in their content and only 29% cases (20 of 70) discussed the importance of multidisciplinary approach to patient management.

In the equity domain, the analysis of the results showed 100% of the cases addressed the patient’s gender and age group. In contrast, 89% case scenarios (62 of 70) failed to include the underserved, disadvantaged, vulnerable populations in the society. Likewise, 77% cases (54 of 70) did not address the ethnicity of the patient, and 53% cases (37 of 70) did not discuss the socioeconomic aspects of the patient. Further, in the cost-effectiveness domain, 91% of the case scenarios (64 of 70) lacked triggers for discussing treatment cost and providing alternatives. Lastly, in the quality domain, 77% cases (54 of 70) did include the concept of person-centred healthcare.

## Discussion

In this study, we looked at the integration of social accountability values such as relevance, quality, equity and cost-effectiveness into the case scenarios (total 70) used in the problem-based learning medical curriculum. Overall, we observed that significant amounts of these social accountability values were embedded through suitable triggers in the case scenarios. However, their consistencies across different units remained varying.

### Relevance

In reference to the relevance, we observed the majority of the case scenarios (73%) addressed the major health problems or social health concerns of the country. It is vital for the medical school to understand and frame its education, training and research directed towards the major health concerns of the community, region, and/or nation they have a mandate to serve [1, 3]. Similarly, 70% of the case scenarios had triggers addressing one or more social determinants of health. Improving our understanding of the social determinants of health such as environments in which people are born, grow, live, work and age, factors such as socioeconomic status, education, employment, and access to health care is critical to provide targeted healthcare and in shaping people’s health [21–23].

A majority of the PBL case scenarios (87%) reflected contextual integration of medical professionalism, evolving roles of doctors in health system (79%) and reflected healthcare referral system based on the case complexity (73%). We believe these traits are critical in providing good clinical care, maintaining good medical practice, relationships with patients, working with colleagues, probity, and health [24]. The medical profession now recognizes the importance of early introduction of professionalism into medical curriculum to provide learning opportunities, gaining experience and reflecting on the values of medical professionalism for better patient care [25].

In the same note, the role of doctor has undergone quantum shifts from the person who knew medicine to a manger, social worker, teacher, advocate, and leader to name a few. The doctors in the twenty-first century should possess the necessary skills for working in teams, utilizing resources effectively, providing patient-centered care, advocating for health care systems, and increasing accessibility for patients. Therefore, knowledge and understanding of the social determinants of health will provide the information and framework to understand the patient’s need and societal factors that are intertwined with health outcomes [26] .

Highlighting the importance of multidisciplinary approach to patient management, we observed only 29% case scenarios had successfully integrated it; similarly, around 49% cases had integrated health system management issues into their content and 59% cases had triggers applying values of health promotion/prevention measures in patient management. We feel the lack of triggers in initiating learning outcomes particularly in the above mentioned domains need improvements. It is well known that multidisciplinary care model that brings together different providers such as physicians, nurses, social workers, and other specialists not only improve health care outcomes but also reduce potential for errors [27]. Given the need for doctors to actively collaborate with multiple professionals, it becomes a key necessity to introduce such learning modules early in the medical curriculum. Similarly, the medical students should be educated about the structure of the healthcare system they want to be a part, early in their curriculum. This will help them to integrate into the nation’s healthcare system and improve their understanding of healthcare legislations, concerns and issues within the healthcare system [28].

### Equity

In the equity domain of social accountability, the case scenarios used in our problem-based learning curriculum showed complete integration in addressing patient’s gender and age group. In comparison, the case scenarios failed to include underserved, disadvantaged or vulnerable populations in society (89%), patient ethnicity (77%) and address socioeconomic status of the patients in 53% cases. Socioeconomic status (SES) of an individual constituting his education, occupation and income has a significant impact on health and access to healthcare. Low SES is an important determinant of access to health care. People with low SES are at a greater risk of poor health, more likely to remain uninsured, seek healthcare less often and may receive poor-quality healthcare. SES can also affect the lifestyle and life expectancy of the person. Thus SES is a good predictor of health outcomes of an individual [29, 30]. Similarly, ethnicity play a significant role in health and access to healthcare. Racial and ethnic disparities does exit in healthcare and are important determinants of social accountability [31]. In order to sensitize the students about the importance of SES, ethnicity and the identification of vulnerable population, the PBL case scenarios should include these information in patient's profile to trigger suitable discussion and highlight its importance [32].

### Cost-effectiveness

Majority of the case scenarios (91%) needs improvement in triggering discussion about treatment cost and providing alternatives with their patients. While most medical curriculum are good in training the students to discuss the diagnosis, treatment and drug side effects with their patients, they lack in sharing financial issues with them. Empowering the patient in their own healthcare decision-making especially the financial issues such as treatment cost and available treatment alternatives has become an important responsibility of the physician [33]. In order to inculcate these traits, students should be engaged in learning how to disclose the financial consequences and suitable alternatives with their patients through the modelled case scenarios.

### Quality

Lastly, 78% PBL case scenarios used in our curriculum includes the concept of person-centred healthcare, thereby making sure that the patient and the doctor consulted the patient about his health needs, and values when making clinical decisions [34].

## Limitation

One of the limitation of the current study is that we have performed quantitative analysis of the PBL case scenarios for the integration of social accountability values in them. We believe further qualitative analysis of these values in the PBL case scenarios will generate corroborative rich textual information.

Moreover, comparing the applications of social accountability values in PBL cases scenarios across different units such as cardiovascular, musculoskeletal, neurosciences, renal and reproductive etc reported some degree of variability. These variations might be due to the nature of the unit content. For example, the PBL cases scenarios used in endocrine and haematology systems are essential part of learning human physiology and pathology, but lacked in reflecting the application of social accountability values. However, authors believe with careful reviews and regular updates, it is possible to inject triggers of social accountability values into the learning materials without jeopardizing their fundamental core objectives.

## Conclusions

In conclusion, this study is first of its kind that has analysed the integration of social accountability values in the case scenarios used in a problem-based learning medical curriculum. The social accountability values addressing the major social health concerns of UAE, social determinants of health, contextual integration of medical professionalism, evolving roles of doctors in the health system, healthcare referral system based on the case complexity, involvement of different stakeholders in healthcare and dealing with the psychosocial issues rather than only disease-oriented issues were effectively integrated in the majority of the case scenarios.

On the other hand, the social accountability values related to the importance of multidisciplinary approach to patient management, the health system management issues, the values of health promotion/prevention, consideration to the underserved, disadvantaged or vulnerable populations in society, the socioeconomic status of patients, ethnicity, and cost-effectiveness were addressed inadequately in the case scenarios.

Medical educators can use this valuable data to calibrate their curriculum content especially those using problem-based learning curriculum to integrate the values of social accountability such as relevance, quality, equity and cost-effectiveness into their curriculum. On a similar note, it is essential that the leaders in healthcare fully understand and appreciate the perspective of all their community needs and stakeholders demands, so that, together they can prepare the next generation healthcare providers that will be ready to serve the ever changing and diverse needs of the very society to which they belong [35, 36].

## Data Availability

The original data can be acquired from the corresponding author upon reasonable request through email.
